# Case Report: A Relatively Mild Presentation of Unilateral Congenital Pulmonary Lymphangiectasia

**DOI:** 10.3389/fped.2021.657473

**Published:** 2021-04-22

**Authors:** Dionne Adair, Raja Rabah, Maria Ladino-Torres, Thomas G. Saba

**Affiliations:** ^1^Department of Pediatrics, CS Mott Children's Hospital, Michigan Medicine, Ann Arbor, MI, United States; ^2^Department of Pathology, CS Mott Children's Hospital, Michigan Medicine, Ann Arbor, MI, United States; ^3^Department of Radiology, CS Mott Children's Hospital, Michigan Medicine, Ann Arbor, MI, United States

**Keywords:** lymphangiectasia, pediatric, histology, computed tomography, case report

## Abstract

Pulmonary lymphangiectasia (PL) is a rare congenital disorder of pulmonary lymphatic development. Although it was traditionally a fatal disorder of infancy, some cases in later childhood have been reported, suggesting a spectrum of severity. We present an unusual case of unilateral, congenital pulmonary lymphangiectasia. Our patient presented with neonatal respiratory distress, a chronic wet cough and recurrent episodes of bronchitis. Chest CT revealed thickening of the interlobular septae of the right lung. A lung biopsy confirmed the diagnosis of lymphangiectasia. His clinical course is characterized by chronic coughing and recurrent bronchitis but normal growth and development. This case illustrates a relatively mild presentation of unilateral PL, which, along with other reports, suggests variability in the presentation and severity of this disorder. In the absence of imaging and histological examination, mild presentations may go undiagnosed.

## Introduction

Pulmonary lymphangiectasia (PL) is a rare disorder characterized by dilation of lymphatic vessels in the lung. This condition is predominantly seen in infancy and has traditionally carried a poor, and often, fatal prognosis for neonatal-onset cases (1, 2). We report a case of unilateral congenital pulmonary lymphangiectasia presenting with relatively mild symptoms.

## Case Description

The patient was born at term *via* cesarean section. He required continuous positive airway pressure (CPAP) and oxygen supplementation for <24 h after birth and was diagnosed with transient tachypnea of the newborn. Shortly after discharge from hospital, at the age of 2 weeks, he began to have a daily wet cough and nasal congestion. The cough was unresponsive to short-acting beta agonists but improved with antibiotics. He was re-admitted to the hospital for bronchiolitis at 1 month of age. A chest radiograph at the time revealed asymmetrical increased reticular opacities on the right lung. He presented to the Pediatric Pulmonology clinic at 4 months of age with persistent daily coughing but otherwise normal growth and development. On physical examination, he was a well-appearing infant. His respiratory exam revealed no crackles, wheezing, or tachypnea and normal oxyhemoglobin saturations in room air. He had no physical exam features consistent with Trisomy 21 or Noonan's syndrome. The patient's older brother has cystic fibrosis. Sweat testing on the patient was negative. Genetic testing did not identify bi-allelic disease-causing mutations and a chromosomal microarray was normal. He underwent flexible fiberoptic bronchoscopy which revealed bilateral small, edematous airways. The bronchoalveolar lavage fluid contained predominantly histiocytes with negative cultures and no lipid-laden macrophages with Oil Red O stain. Ciliary ultrastructure was normal under transmission electron microscopy. Further testing revealed normal immunoglobulins and vaccine titers and a normal video fluoroscopic swallow evaluation.

A throat culture at the age of one was positive for *Pseudomonas aeruginosa*. In an effort to overcome a subacute bronchitis refractory to multiple courses of antibiotics, antibiotic coverage was re-directed toward *Pseudomonas aeruginosa*.

**Figure 1:**
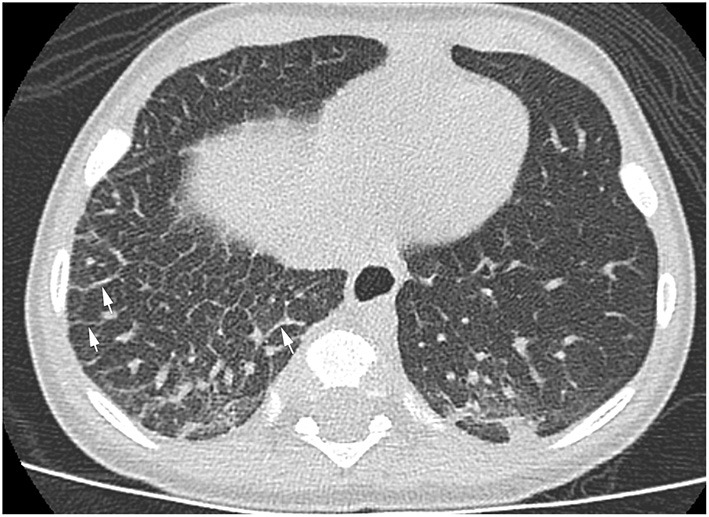
High resolution chest CT showing diffuse thickening of the interlobular septae (white arrows) in the right lung.

**Figure 2:**
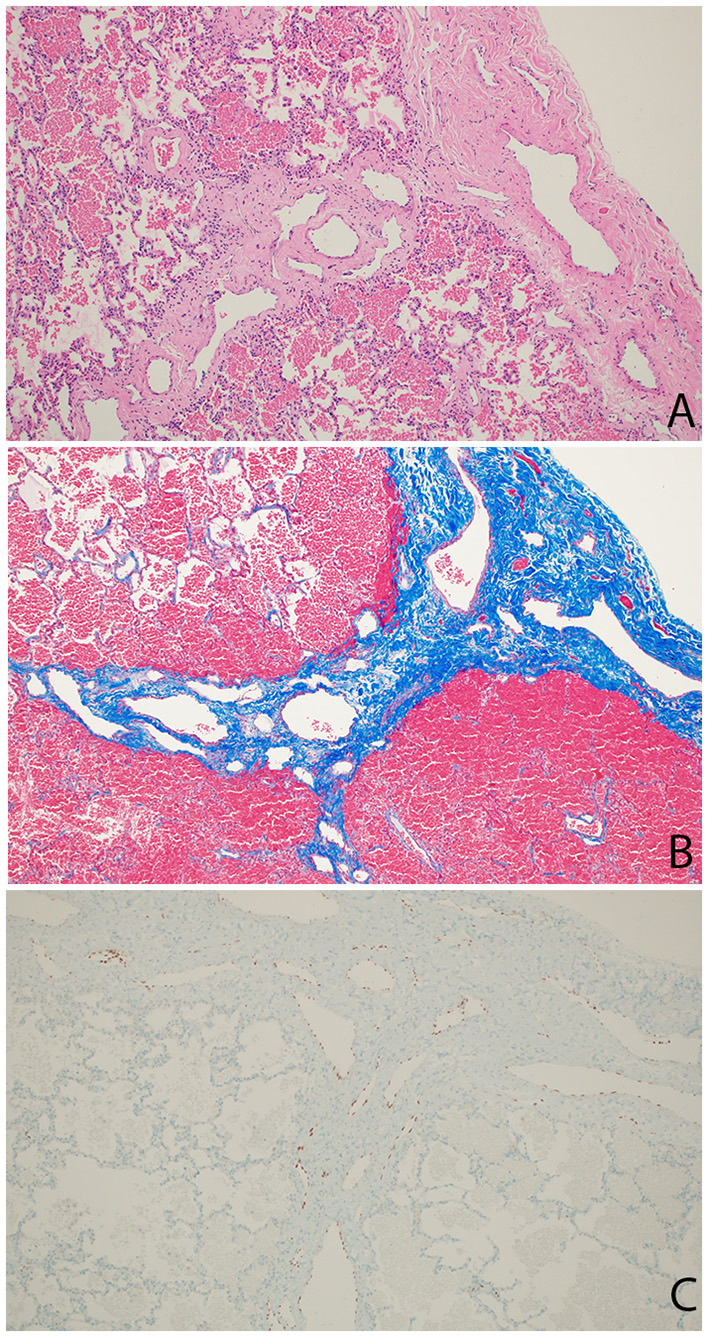
Wedge resection of right lower lobe. **(A)** Hematoxylin-Eosin stain showing thickened pleura and septum containing increased number of markedly dilated lymphatic channels. **(B)** Trichrome stain showing fibrosis (blue) in the pleura and septum. **(C)** Immunostain staining nuclei red confirming lymphatic origin of endothelium of dilated pleural and septal vessels.

The patient continued to have several presentations to the emergency room with viral-induced coughing exacerbations and wheezing on examination. He was treated with short acting beta agonists, systemic steroids and antibiotics resulting in some symptomatic relief. Chest radiographs persistently showed right-sided reticular opacities. Due to the patient's steroid- and bronchodilator-responsive clinical picture, he was started on daily inhaled corticosteroids. An echocardiogram revealed normal anatomy without evidence of venous obstruction or signs of elevated right heart pressures. A high resolution chest CT revealed diffuse, extensive thickening of the interlobular septae on the right leading to a suspicion for unilateral lymphangiectasia (Figure 1). The patient underwent a thoracoscopic lung biopsy at the age of 2 which identified pleural and septal fibrosis with dilated lymphatics consistent with pulmonary lymphangiectasia (Figure 2).

Presently, at age 4, the patient continues to have a chronic cough and recurrent bronchitis requiring prolonged courses of systemic antibiotics (14–21 days) and steroids for symptomatic relief. His respiratory regimen includes airway clearance and inhaled steroids for bronchial hyperreactivity. Although his chronic symptoms and frequent exacerbations have placed a significant burden on his quality of life, his family has committed to excellent treatment adherence and he has maintained normal growth and development. Although modified diets and Octreotide have been used among children with some congenital abnormalities, such as chylothorax, no evidence supports these interventions among children with PL.

## Discussion

Congenital pulmonary lymphangiectasia is a rare condition usually leading to death in the neonatal period. It is characterized by dilated lymphatic channels draining the interstitial and subpleural spaces of the lung. The earliest classification of this disorder by Noonan et al. described three forms of the disorder: isolated congenital pulmonary lymphangiectasia, congenital pulmonary lymphangiectasia associated with pulmonary venous obstruction, and congenital pulmonary lymphangiectasia associated with a generalized defect in lymphatic development (1). Although typically a diffuse disease, unilateral and unilobar cases have been reported (3). There appears to be a male predominance.

The etiology of pulmonary lymphangiectasia is unknown. Theories involve failed regression of large lymphatic channels which appear between 9 and 16 weeks gestation or bronchomediastinal lymphatic obstruction. Genes involved in lymphangiogenesis, such as vascular endothelial growth factors (*VEGF-C, VEGF-D*) and their receptors (*VEGFR-2, VEGFR-3*) have also been implicated (2). There is a well-known association with trisomy 21, Noonan's Syndrome and Turners Syndrome. Cases in multiple family members also suggest a genetic predisposition.

Prenatally, PL may manifest as pleural effusions and hydrops. At birth, patients may have severe distress requiring mechanical ventilation. Traditionally, neonatal-onset cases were fatal. More recently, presumably due to advances in neonatal care, a larger proportion of patients are surviving to infancy. Presentations beyond infancy have been diagnosed with histology. Patients presenting in childhood tend to have more localized disease and a better prognosis. Adult cases with progressive dyspnea and hemoptysis have been reported (4).

Chest radiographic findings include prominent interstitial markings, hyperinflation, ground glass opacities especially in neonates and, less commonly, chylous pleural effusions. Computed tomography (CT) of the chest might show diffuse or segmental interlobular septal thickening, perihilar infiltrates, ground glass opacities and hyperinflation. These findings, when combined with pleural effusions, increase the likelihood of PL (5). Lung biopsy with histology and immunochemical staining demonstrating dilated lymphatic channels and thickening of the interlobular septa and the subpleural space is the gold standard for diagnosis. The findings of interstitial emphysema may appear similarly (6) and lymphatic dilation can sometimes be seen as an artifact associated with the lung biopsy. Histological specimens, therefore, should be analyzed by an experienced pathologist. An echocardiogram should be performed in order to rule out pulmonary venous obstruction such as hypoplastic left heart syndrome and TAPVR causing secondary lymphangiectasia (7). Lymphangiograms have been used in some patients to evaluate for thoracic duct disruption and lymphatic duct dilation (8).

The natural course in survivors is variable. Most patients have chronic cough and wheeze, variable response to short acting beta agonists and an increased propensity for respiratory infections. Bronchoalveolar lavage cultures in patients with PL have identified a number of bacterial pathogens including *Pseudomonas aeruginosa* (9). Neutrophilic bronchitis and copious secretions are common. Although there tends to be chronic respiratory symptoms early in life, many survivors become asymptomatic by school age (9). Treatment, therefore, is patient-specific and mostly supportive. Emerging therapies with short-term benefits include lymphatic embolization and sirolimus, a mammalian target of rapamycin (mTOR) inhibitor known to have antiangiogenic properties (2). Lobectomy or pneumonectomy may be considered in severe cases of localized disease.

## Conclusion

Chronic cough is a common complaint in children. When the most likely causes, such as asthma, infections, post nasal drip and gastroesophageal reflux disease are ruled out, rare conditions must be considered. These include aspiration syndromes, cystic fibrosis, primary ciliary dyskinesia, primary immunodeficiencies, and other rare conditions. This case illustrates an unusual presentation of unilateral congenital pulmonary lymphangiectasia in an infant with chronic, but, relatively mild symptoms, suggesting a wide range of clinical presentations of a traditionally severe disorder. In contrast to patients with more generalized forms of pulmonary lymphangiectasia, affected individuals with more localized findings such as our patient are likely to have a good prognosis. Although there is no cure for this disorder, patients who survive infancy can be managed with supportive therapies.

## Data Availability Statement

The original contributions presented in the study are included in the article/supplementary material, further inquiries can be directed to the corresponding author.

## Author Contributions

DA evaluated and cared for the patient, collected all relevant clinical information and diagnostic testing results, performed the literature review, and wrote the manuscript. RR performed histological examination and description. ML-T provided interpretation of radiological images. TS provided mentorship to DA and oversaw the project. All authors read and approved the manuscript.

## Conflict of Interest

The authors declare that the research was conducted in the absence of any commercial or financial relationships that could be construed as a potential conflict of interest.
